# Identification and validation of hypoxia-derived gene signatures to predict clinical outcomes and therapeutic responses in stage I lung adenocarcinoma patients

**DOI:** 10.7150/thno.56202

**Published:** 2021-03-05

**Authors:** Run Shi, Xuanwen Bao, Kristian Unger, Jing Sun, Shun Lu, Farkhad Manapov, Xuanbin Wang, Claus Belka, Minglun Li

**Affiliations:** 1 Department of Radiation Oncology, University Hospital, LMU Munich, Munich D-81377, Germany.; 2 Department of Medical Oncology, The First Affiliated Hospital, College of Medicine, Zhejiang University, Hangzhou 310003, China.; 3 Research Unit Radiation Cytogenetics, Helmholtz Center Munich, German Research Center for Environmental Health GmbH, Neuherberg D-85764, Germany.; 4 Department of Radiotherapy, Sichuan Cancer Hospital, School of Medicine, University of Electronic Science and Technology of China, Chengdu 610041, China.; 5 Laboratory of Chinese Herbal Pharmacology, Oncology Center, Renmin Hospital, Hubei Key Laboratory of Wudang Local Chinese Medicine Research, Hubei University of Medicine, Shiyan 442000, China.

**Keywords:** Stage I lung adenocarcinoma, Hypoxia, Clinical outcomes, Genomic alterations, Machine learning.

## Abstract

**Rationale:** The current tumour-node-metastasis (TNM) staging system is insufficient for precise treatment decision-making and accurate survival prediction for patients with stage I lung adenocarcinoma (LUAD). Therefore, more reliable biomarkers are urgently needed to identify the high-risk subset in stage I patients to guide adjuvant therapy.

**Methods:** This study retrospectively analysed the transcriptome profiles and clinical parameters of 1,400 stage I LUAD patients from 14 public datasets, including 13 microarray datasets from different platforms and 1 RNA-Seq dataset from The Cancer Genome Atlas (TCGA). A series of bioinformatic and machine learning approaches were combined to establish hypoxia-derived signatures to predict overall survival (OS) and immune checkpoint blockade (ICB) therapy response in stage I patients. In addition, enriched pathways, genomic and copy number alterations were analysed in different risk subgroups and compared to each other.

**Results:** Among various hallmarks of cancer, hypoxia was identified as a dominant risk factor for overall survival in stage I LUAD patients. The hypoxia-related prognostic risk score (HPRS) exhibited more powerful capacity of survival prediction compared to traditional clinicopathological features, and the hypoxia-related immunotherapeutic response score (HIRS) outperformed conventional biomarkers for ICB therapy. An integrated decision tree and nomogram were generated to optimize risk stratification and quantify risk assessment.

**Conclusions:** In summary, the proposed hypoxia-derived signatures are promising biomarkers to predict clinical outcomes and therapeutic responses in stage I LUAD patients.

## Introduction

Lung adenocarcinoma (LUAD) has become the most common subtype of non-small cell lung cancer (NSCLC), a leading cause of cancer death worldwide 1. Unfortunately, even stage I lung cancer has a poor prognosis with about 70% 5-year overall survival after surgical resection 2, revealing the need of treatment escalation, for example with adjuvant therapy. Although adjuvant chemotherapy proved to be beneficial for stage II-III NSCLC patients, its effectivity in stage I remains controversial 3-5. Several large randomized studies failed to demonstrate a significant survival benefit in stage I NSCLC patients 3, 4. Only in an exploratory analysis, Strauss et al. could show a significant benefit for adjuvant chemotherapy in stage I patients with tumour size larger than 4 cm 4. However, without taking distinct tumour biological characters into account, tumour size alone cannot be an optimal criterion for precise risk stratification and decision-making of adjuvant treatment. Therefore, a novel method to identify the high-risk subset of stage I patients who mostly benefit from adjuvant therapy will bring immense value for personalized cancer care 6.

In the recent years, an increasing number of studies have proposed genomic signatures for risk stratification and survival prediction in NSCLC patients 7-9. However, due to some problematic issues such as limited sample size, individual heterogeneity and technical bias in these studies, most prognostic signatures lacked reproducibility and few of them were applied to clinical routine practice 10.

Hypoxic environment is a result of imbalance between oxygen demand and supply, and intratumoral hypoxia is a critical hallmark of cancer which is widely associated with malignant progression, therapeutic resistance and poor prognosis 11-13. In our previous study, we established a hypoxia-related gene signature to predict prognosis in stage I-II LUAD patients 14. However, the clinical outcomes, oncogenic pathways, genomic alterations and therapeutic responses underlying different hypoxic conditions remain obscure in absolute early-stage (stage I) patients, a group who urgently need reliable biomarkers to guide adjuvant therapy.

In the present study, hypoxia was identified as a dominant risk factor for overall survival in stage I LUAD. A series of bioinformatic and machine learning approaches were jointly used to screen for robust candidate genes and to build individualized hypoxia-derived signatures to predict overall survival (OS) and immune checkpoint blockade (ICB) therapy response for stage I patients, respectively.

## Materials and methods

### Data acquisition

We retrospectively analysed the gene expression profiles and clinical parameters of primary LUAD patients from 14 public cohorts, including 13 microarray datasets and 1 RNA-Seq dataset from The Cancer Genome Atlas (TCGA). Only patients meet the following two criteria were included: i) detailed TNM staging information includes stage I, IA, IB or T1N0M0 and T2aN0M0; ii) overall survival information includes follow-up time and vital status. Overall, a total of 1,400 stage I patients were included in our study. The dataset GSE72094 was used as the training set because it is an independent microarray dataset with an appropriate sample size 15. Raw CEL files from a same chip platform (Affymetrix HG-U133A or U133 Plus 2.0) were downloaded and integrated to a new cohort using a robust multi-array average (RMA) method, with Combat algorithm eliminating the batch effects 16, 17. The first validation set was composed of four independent microarray datasets (GSE68465 18, GSE14814 19, GSE31547 [unpublished], Chitale's cohort 20) produced from U133A, and the second validation set was composed of six datasets (GSE30219 21, GSE31210 22, GSE50081 23, GSE37745 24, GSE29013 25, E-MTAB-923 26) from U133 Plus 2.0. Moreover, another three datasets from different platforms were used as three independent validation sets including TCGA, GSE41271 27 and GSE13213 28. The details of clinicopathological features in each cohort were summarized in Figure S1. In addition, transcriptome data and therapeutic responses of 20 BALB/c mice inoculated subcutaneously with AB1-HA cells received anti-CTLA-4 treatment were acquired from GSE63557 29. In microarray analysis, probe IDs were mapped to gene symbols according to the corresponding annotation file, and expression measurements of all probes related to a same gene were averaged to obtain a single value.

The somatic mutation and copy number variation (CNV) profiles were obtained from TCGA data portal (https://portal.gdc.cancer.gov/). Somatic mutation data, which are sorted in the form of Mutation Annotation Format (MAF), were analysed using R package 'maftools'. Significant amplifications or deletions of copy number were detected using GISTIC 2.0 with a threshold of FDR *Q* < 0.05.

### Transcriptome profile analysis of tissues, single cells and cell lines

Transcripts per million (TPM) value of transcriptome of 69 LUAD cell lines was obtained from Cancer Cell Line Encyclopedia (CCLE) 30. In addition, we analysed a microarray data (GSE30979) of LUAD sample fragments which were cultured ex vivo under hypoxia (1% oxygen) or normoxia for three days 31. Single-cell RNA-Seq data of 77 cells derived from a LUAD patient without any treatment was accessed from GSE69405 32. All the microarray and RNA-Seq data included were normalized and log2 transformed.

### Selection of candidate genes and establishment of hypoxia-related signatures

The levels of cancer-related hallmarks raised by Hanahan and Weinberg 33 such as “Cell cycle progression (CCP)”, “Epithelial-mesenchymal transition (EMT)” and “Hypoxia” in each sample from the training set were quantified using a single-sample gene set enrichment analysis (ssGSEA) algorithm based on the transcriptome profiling data and corresponding gene sets retrieved from Molecular Signatures Database (MSigDB), and the gene set of stemness was obtained from a previously published literature 34-36. CIBERSORT was used to quantify immune infiltration based on the transcriptome profiling data of each sample in the training set 37. Z-score scaling was applied to both ssGSEA and immune infiltration scores. Weighted gene co-expression network analysis (WGCNA) was used to construct a scale-free co-expression network using the R package 'WGCNA' and to identify a gene module which is mostly correlated with hypoxia 38. On the other hand, potential targets of HIF1A in LUAD were obtained from Cistrome Cancer which integrated analysis of TCGA molecular profiling data and public transcription factor ChIP-Seq profiles 39. Stage I LUAD-specific hypoxia-related candidates were identified from the intersection of “Hypoxia-related module” and “HIF1A targets”. The least absolute shrinkage and selection operator (LASSO) Cox or logistic regression models and random forest (RF) algorithm were used to further screen for the most robust candidates 40. Finally, a hypoxia-related prognostic risk score (HPRS) and a hypoxia-related immunotherapeutic response score (HIRS) for each sample were calculated as follows:


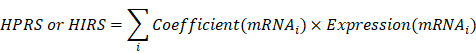


### Additional bioinformatic and statistical analyses

IBM SPSS Statistics 20 (IBM Corp., Armonk, N.Y., USA), GraphPad Prism 8.0 (GraphPad Software Inc, San Diego, CA), Stata 12 (StataCorp LLC, Texas, USA) and R software (version 3.6.0, http://www.r-project.org) were used to analyse data and plot graphs. Distance between different hallmarks was depicted using hierarchical clustering analysis. Multivariate Cox regression analysis was performed to evaluate the risk significance of each variable for overall survival. Non-negative matrix factorization (NMF) consensus clustering (R package 'NMF') was used to obtain clusters based on a gene expression matrix. Principal component analysis (PCA) was used to visualize dissimilarity of two groups using R package 'pca3d', and the percentages of explained variances were calculated using R package “factoextra”. The Kaplan-Meier method was used to draw survival curves, and the log-rank test was performed to evaluate survival difference. Random-effects meta-analysis model was used to calculate a pooled hazard ratio (HR). Time-dependent concordance index (C-index) and time-dependent receiver operating characteristic (tROC) analysis were used to compare the predictive capacity of survival among different variables with R packages 'survConcordance' and 'survivalROC'. Survival net benefits of each variable were estimated with decision curve analysis (DCA) using 'stdca.R'. Recursive partitioning analysis was performed to construct a survival decision tree for risk stratification with R package 'rpart'. A nomogram and calibration curve were plotted using R package 'rms'. Differentially expressed genes (DEGs) were identified with a threshold of FDR q < 0.0001 based on reads count matrix and R package “DESeq2”, and submitted for Gene Ontology enrichment analysis using Metascape 41. The R package 'pRRophetic' was applied to estimate the chemotherapeutic responses in the training cohort. Potential ICB therapy response was predicted with tumour immune dysfunction and exclusion (TIDE) algorithm 42, and the RF algorithm was used to screen for the most important candidates associated with ICB therapy response with two parameters 'mtry' and 'ntree' of optimal values. Student's t-test or one-way analysis of variance was used to analyse differences between groups in variables with a normal distribution. Categorical variables between two groups were compared using chi-square test. *P* value < 0.05 was considered statistically significant.

## Results

### Schematic diagram of the study design

**Figure 1:**
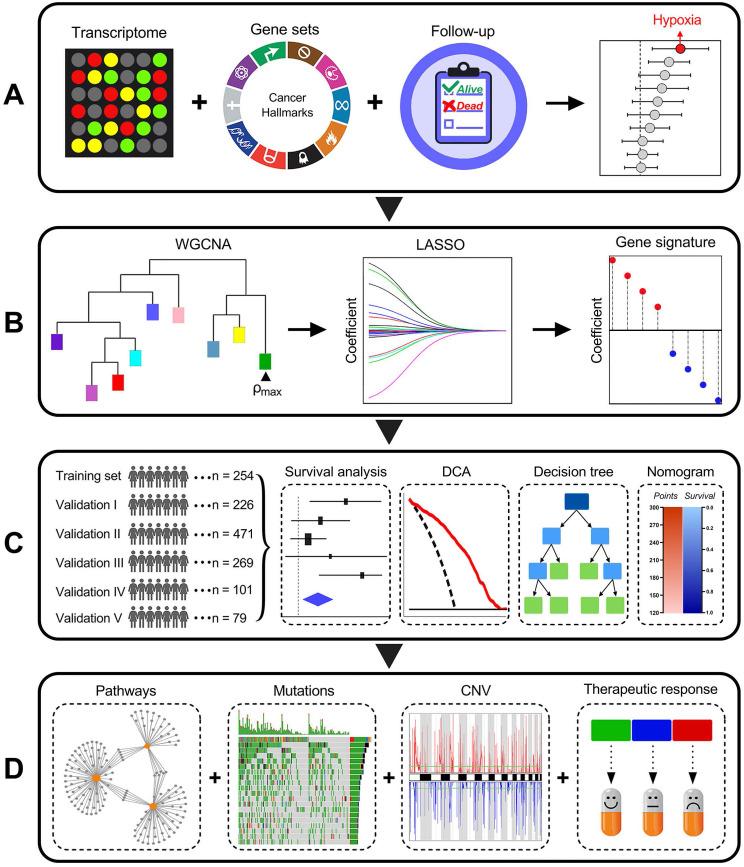
** Schematic diagram of the study design.** (A) Among various hallmarks of cancer, hypoxia was identified as the primary risk factor for overall survival in stage I LUAD patients. (B) WGCNA and LASSO Cox algorithms were combined to develop a hypoxia-related gene signature for prognosis. (C) The prognostic and predictive capacities were validated in different cohorts and methods. (D) Comprehensive analyses of enriched pathways, genomic alterations, CNVs and therapeutic responses in different risk groups.

Among various hallmarks of cancer defined by Hanahan and Weinberg 33, hypoxia was identified as the most significant risk factor for overall survival in stage I LUAD patients (Figure 1A). Then, WGCNA and LASSO Cox algorithm were combined to screen for robust hypoxia-related prognostic biomarkers (Figure 1B). Subsequently, the prognostic capacity of the hypoxia-related signature was evaluated in the training cohort and five independent validation cohorts. In addition, meta-analysis was performed to further validate its prognostic power, DCA was used to compare the survival net benefits of each variable, and an integrated decision tree and nomogram were built to improve risk stratification and survival prediction (Figure 1C). Finally, the enriched pathways of DEGs, genomic alterations and therapeutic responses were analysed and compared (Figure 1D).

### Hypoxia was identified as a primary risk factor for overall survival in stage I LUAD

**Figure 2:**
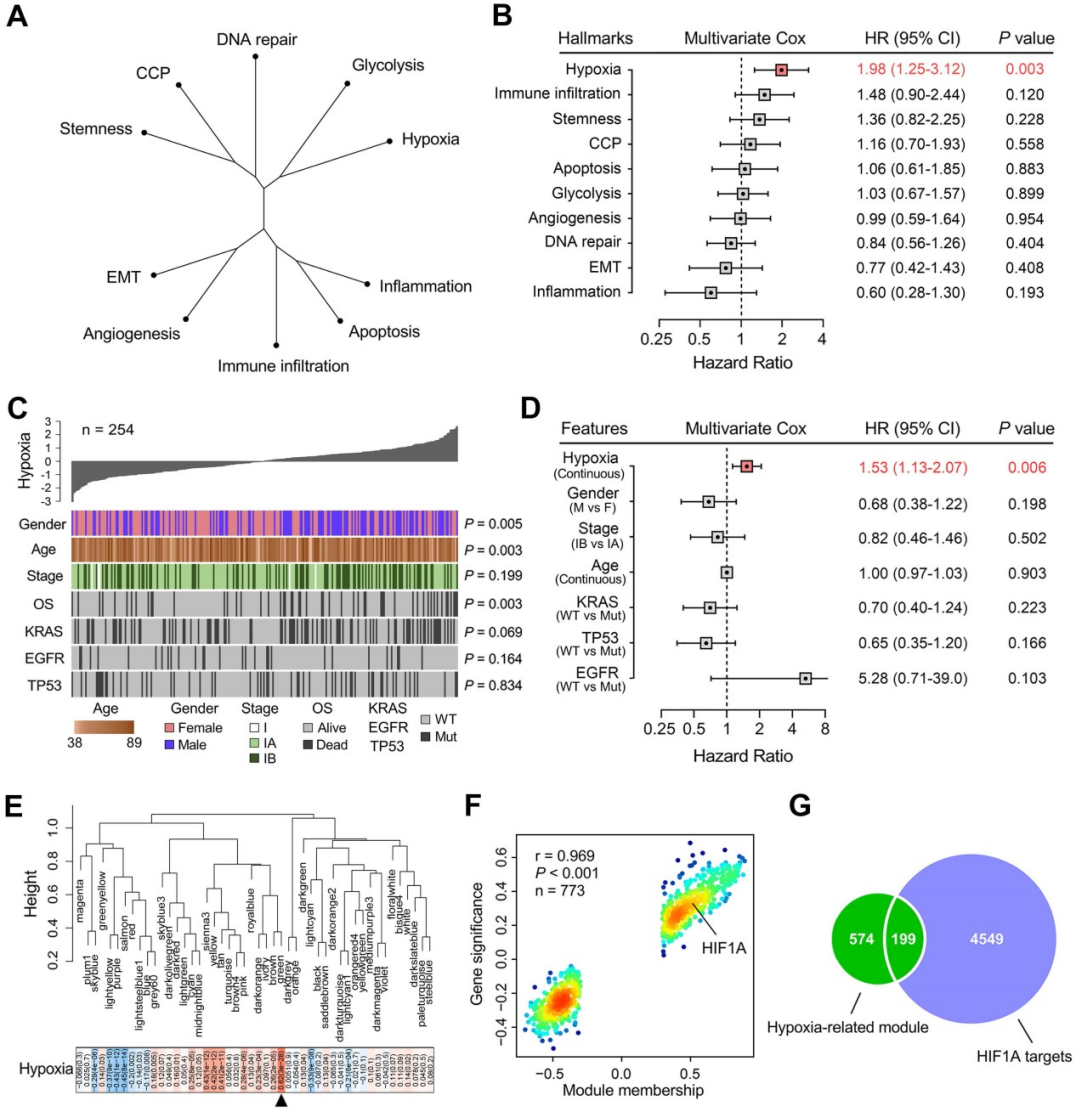
** Hypoxia is a dominant risk factor for stage I LUAD, and a set of 199 stage I-specific hypoxia-related candidates were identified.** (A) An unrooted clustering dendrogram shows the distance between different hallmarks of cancer. (B) Multivariate Cox regression analysis demonstrated that hypoxia was the only significant risk factor for overall survival among various hallmarks of cancer (*P* = 0.003). (C) A heatmap depicts the correlations between hypoxia ssGSEA Z-scores and clinicopathological features and mutations of driver oncogenes. (D) Multivariate Cox regression analysis revealed that hypoxia was the only significant variable for overall survival among clinicopathological features (*P* = 0.006). (E) WGCNA was performed to construct a scale-free co-expression network. The green gene module exhibited the highest correlation with hypoxia (r = 0.62, *P* = 3e-28) and was considered as “hypoxia-related module”. (F) The module membership and gene significance of 773 genes involved in the green module exhibited a highly positive correlation (r = 0.969, *P* < 0.001), and HIF1A was located in the core part which is positively correlated with hypoxia. (G) 199 overlapping candidates were identified in the intersection of “hypoxia-related module” and “HIF1A targets”.

An unrooted clustering dendrogram was generated to show the distance between cancer-related hallmarks based on their Z-score matrix in the training set. We observed that “Hypoxia” and “Glycolysis” remained close to each other but relatively distant to other hallmarks (Figure 2A). Multivariate Cox regression analysis demonstrated that hypoxia was the only significant risk factor for overall survival among various cancer-related hallmarks (*P* = 0.003; Figure 2B). A heatmap was plotted to depict the correlations between hypoxia ssGSEA Z-scores and clinicopathological features and mutations of driver genes in the training cohort, and significant correlations between hypoxia and gender, age and overall survival status were observed (Figure 2C). In addition, multivariate Cox regression analysis revealed that hypoxia was the only significant variable for overall survival among these features (*P* = 0.006; Figure 2D). These findings showed that hypoxia was a dominant risk factor for overall survival in stage I LUAD among cancer hallmarks and clinicopathological features.

### Identification of stage I LUAD-specific candidate genes involved in hypoxia

WGCNA was performed with transcriptome profiling data and hypoxia ssGSEA Z-scores to construct a scale-free co-expression network. A total of 48 gene modules were generated with a power of 5 as the optimal soft threshold (Figure 2E & Figure S2). Among these modules, the green module exhibited the highest correlation with hypoxia (r = 0.62, *P* = 3e-28) and was considered as “hypoxia-related module” (Figure 2E). The module membership and gene significance of 773 genes involved in the green module exhibited a highly positive correlation (r = 0.969, *P* < 0.001), and we observed HIF1A was located in the core part which is positively correlated with hypoxia (Figure 2F). Considering HIF1A acts as a core transcription factor in hypoxia, we intersected the “hypoxia-related module” with 4,748 potential targets of HIF1A in LUAD and found 199 candidate genes in the intersection (Figure 2G & Table S1), and the 199 candidates were considered as “stage I LUAD-specific hypoxia-related genes”.

**Figure 3:**
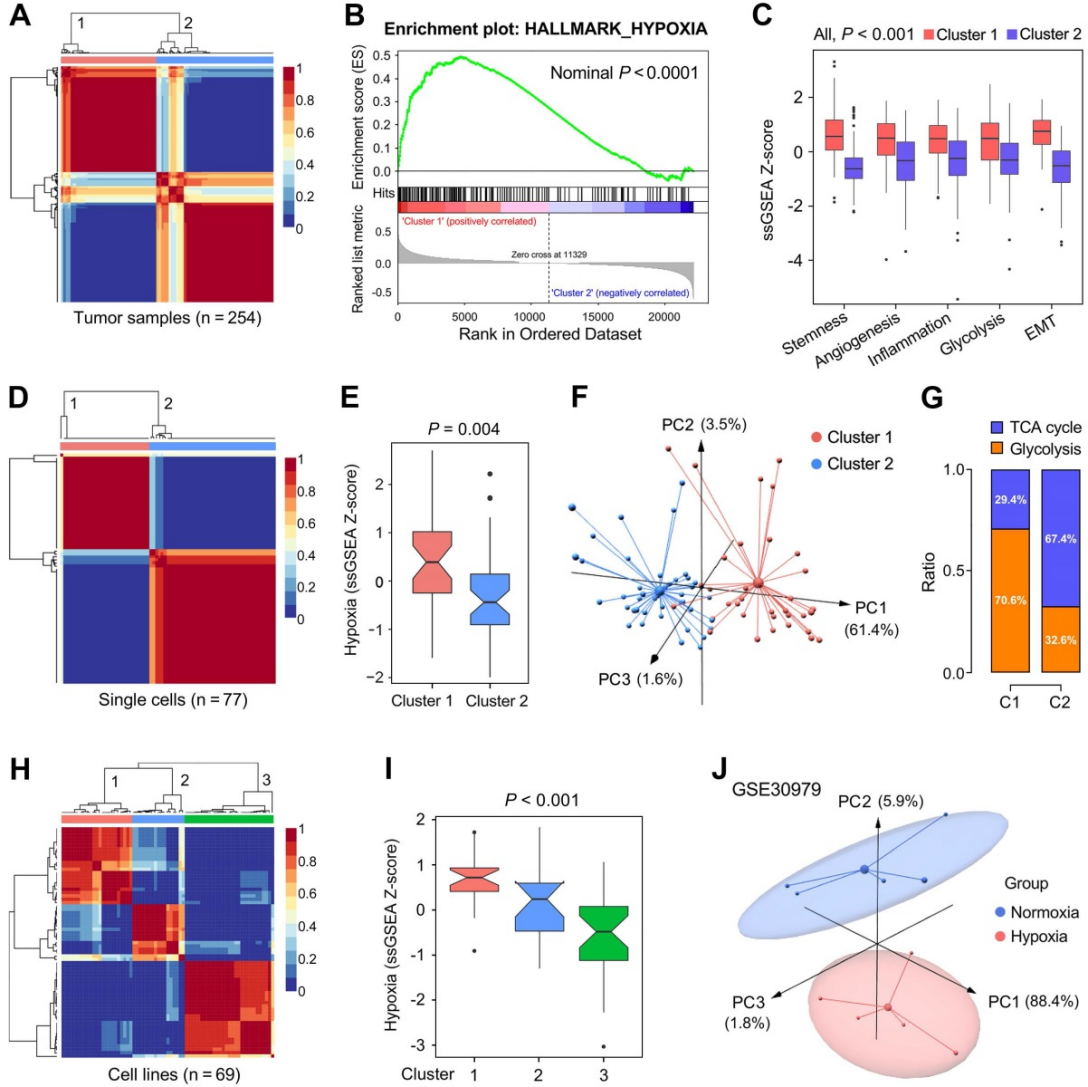
** The representation of the 199 hypoxia-related genes was validated at different levels.** (A) NMF consensus clustering was used to divide 254 training samples into two clusters, and (B) GSEA analysis indicated that cluster 1 exhibited significant hypoxia enrichment. (C) Hypoxia-induced influences on stemness, angiogenesis, inflammation, glycolysis and EMT. (D & E) 77 single cells derived from a same LUAD patient were divided into two clusters with different hypoxia level (*P* = 0.004). (F) The two clusters exhibited absolute dissimilarity in the PCA analysis and (G) different distribution ratio of glycolysis and TCA cycle. (H) 69 LUAD cell lines from CCLE were divided into three clusters, and (I) hypoxia levels were progressively decreased in the three clusters (*P* < 0.001). (J) PCA analysis demonstrated that samples cultured ex vivo under hypoxia or normoxia were clearly separated into two discrete groups with the 199 genes expression matrix.

We further validated the 199 candidate genes as robust hypoxia-related genes in three aspects: LUAD tissues, single cells and cell lines. Firstly, NMF was used to divide 254 training samples into two clusters based on the expression profiles of the 199 genes with an optimal k of 2 (Figure 3A), and GSEA analysis indicated that cluster 1 exhibited significant hypoxia enrichment compared to cluster 2 (Figure 3B). Boxplots showed the ssGSEA scores of some critical hallmarks including stemness, angiogenesis, inflammation, glycolysis and EMT were significantly elevated in cluster 1 (All, *P* < 0.001; Figure 3C). Similarly, with an optimal k of 2, 77 single cells derived from a same LUAD patient were divided into two clusters (Figure 3D) with different hypoxia level (*P* = 0.004; Figure 3E). Furthermore, the two clusters exhibited absolute dissimilarity in the PCA analysis (Figure 3F) and different distribution ratio of glycolysis and TCA cycle (Figure 3G). With an optimal k of 3, 69 LUAD cell lines from CCLE were divided into three clusters based on the log_2_TPM matrix of the 199 genes (Figure 3H), and hypoxia levels were progressively decreased in the three clusters (*P* < 0.001; Figure 3I). Finally, PCA analysis demonstrated that samples cultured ex vivo under hypoxia or normoxia were clearly separated into two distinct groups with the 199 genes expression matrix (Figure 3J).

### Establishment and validation of a hypoxia-related prognostic signature for overall survival in stage I patients

**Figure 4:**
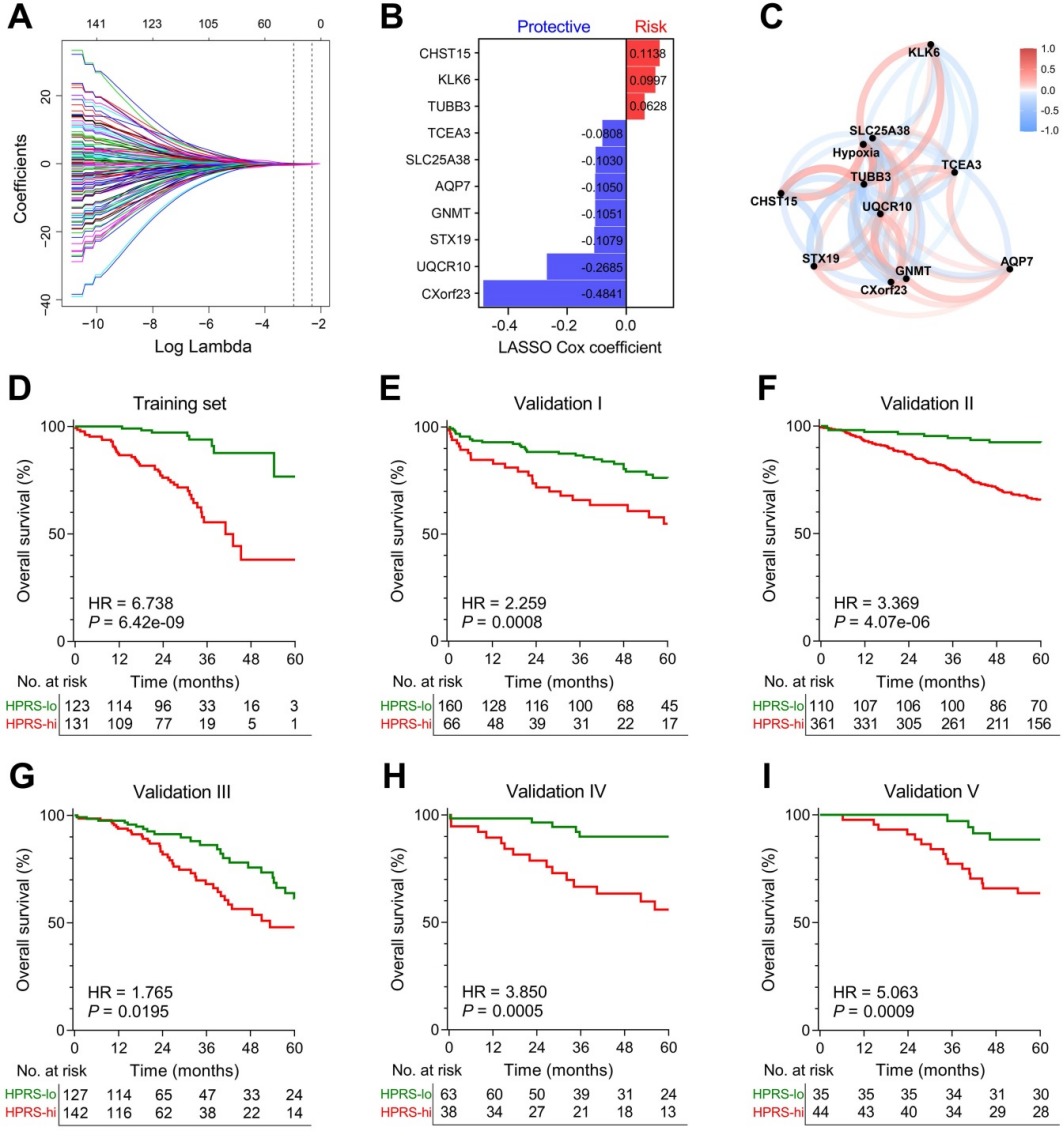
**Establishment and validation of a prognostic hypoxia signature for stage I patients.** (A) LASSO Cox regression algorithm was used to identify the most robust prognostic genes. (B) An ensemble of 10 genes remained with individual coefficients. (C) A correlation network involving the 10 genes and hypoxia in the training cohort. (D) Kaplan-Meier analysis demonstrated that patients with higher HPRS exhibited worse overall survival in the training cohort, and (E-I) the prognostic value was validated in five independent cohorts.

LASSO Cox algorithm was used to identify the most robust prognostic genes among the 199 candidate genes. 10-fold cross-validation was applied to overcome over-fitting effect, and an optimal λ value of 0.051 was selected (Figure 4A & Figure S3). An ensemble of 10 genes remained with individual coefficients (Figure 4B), which were integrated to build a hypoxia-related prognostic signature. A correlation network involving the 10 genes and hypoxia ssGSEA Z-scores in the training cohort was shown in Figure 4C. Using the established formula, a hypoxia-related prognostic risk score (HPRS) for each sample was calculated and normalized to Z-score in each cohort. As shown in Figure 4D, Kaplan-Meier analysis demonstrated that patients with higher HPRS exhibited worse overall survival in the training cohort (HR = 6.738, 95% CI = 3.902-11.64, *P* = 6.42e-09). Then, the prognostic value of HPRS was validated in five independent cohorts (Validation I: HR = 2.259, 95% CI = 1.309-3.899, *P* = 0.0008; Validation II: HR = 3.369, 95% CI = 2.331-4.869, *P* = 4.07e-06; Validation III: HR = 1.765, 95% CI = 1.082-2.880, *P* = 0.0195; Validation IV: HR = 3.850, 95% CI = 1.771-8.369, *P* = 0.0005; Validation V: HR = 5.063, 95% CI = 2.311-11.09, *P* = 0.0009; Figure 4E-I).

### Comparison of prognostic and predictive capacities between HPRS and traditional features

**Figure 5:**
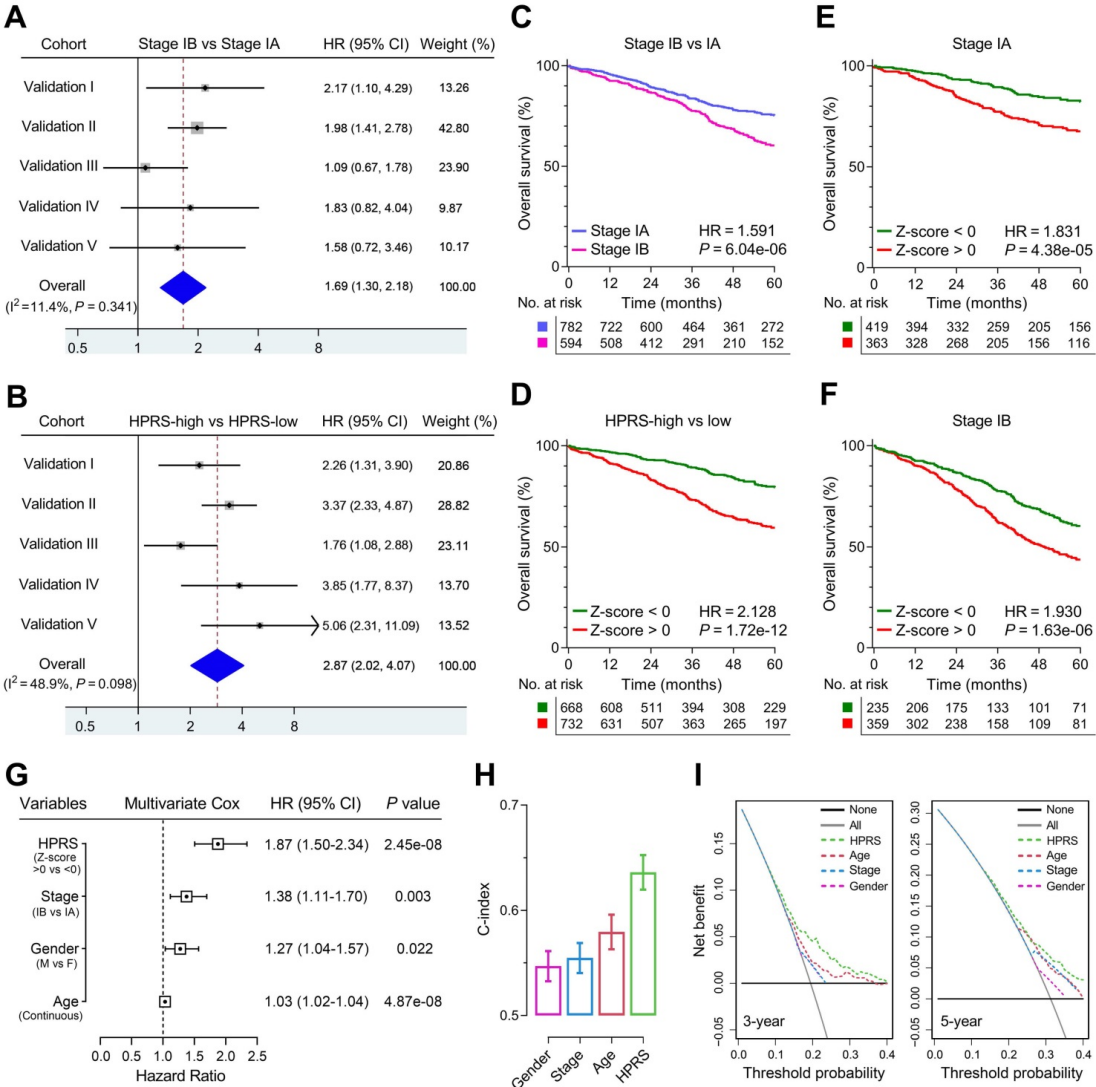
** Comparison of the prognostic and predictive capacities between HPRS and traditional features.** (A & B) Meta-analysis was performed to calculate the pooled HR of TNM staging classification or HPRS, respectively. (C & D) Kaplan-Meier analysis was used to plot survival curves and evaluate survival difference in the pooled cohort to visualize the prognostic values of staging classification and HPRS, respectively. (E & F) In both stage IA and stage IB subgroups, HPRS retained its prognostic capacity to discriminate high-risk subset with Z-score of zero as a cut-off value. (G) Multivariate Cox regression analysis was performed on four variables including HPRS, p-stage, gender and age in the pooled cohort. (H) C-index of HPRS ranks first among all the parameters. (I) DCA graphically illustrated that HPRS brought more net benefit of survival than other parameters at two different time points.

Meta-analysis was performed to calculate the pooled HR of TNM staging classification or HPRS with the exclusion of the training set, respectively. In comparison, staging classification exhibited a pooled HR of 1.69 (95% CI = 1.30-2.18; Figure 5A), while HPRS showed a pooled HR of 2.87 (95% CI = 2.02-4.07; Figure 5B). In addition, Kaplan-Meier analysis was used to plot survival curves and evaluate survival difference in the pooled cohort to visualize the prognostic values of staging classification and HPRS, respectively (Figure 5C & D). In both stage IA and stage IB subgroups, HPRS retained its prognostic capacity to discriminate high-risk subset with Z-score of zero as a cut-off value (in stage IA: HR = 1.831, *P* = 4.38e-05, Figure 5E; in stage IB: HR = 1.930, *P* = 1.63e-06, Figure 5F). Further, multivariate Cox regression analysis was performed on four variables including HPRS (Z-score of zero as cut-off value), p-stage (IA and IB), gender (male and female) and age (continuous value) in the pooled cohort. We observed that all the four parameters are independent risk factors for overall survival in stage I patients, and HPRS exhibited highest significance (HR = 1.87, 95% CI = 1.50-2.34, *P* = 2.45e-08; Figure 5G) among these variables. In addition, C-index of HPRS ranks first among these variables, which suggests the most powerful predictive capacity (Figure 5H). DCA graphically illustrated that HPRS brought more net benefit of survival than other parameters at two different time points (Figure 5I).

### Construction of integrated models to optimize risk stratification and survival prediction in stage I patients

**Figure 6:**
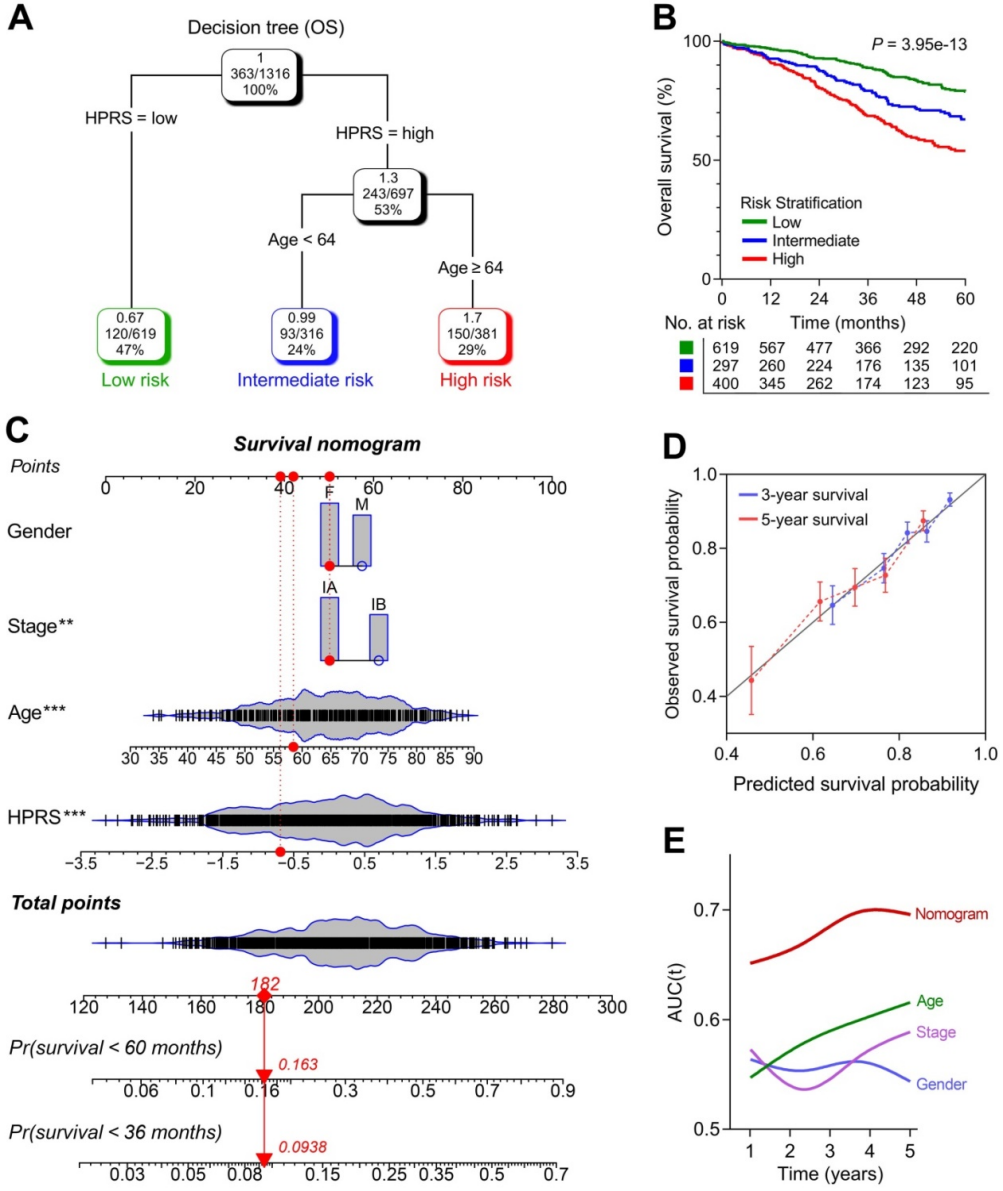
** A survival decision tree and nomogram were generated to improve risk stratification and estimate survival probability.** (A) Patients with full-scale annotations including HPRS, p-stage, gender and age were used to build a survival decision tree to optimize risk stratification. (B) Significant differences of overall survival were observed among the three risk subgroups. (C) Details of the nomogram. (D) The nomogram shows a high accuracy in the calibration analysis. (E) Compared with other clinicopathological features, the nomogram exhibited the most powerful capacity for survival prediction.

1,316 patients with full-scale clinical annotations including p-stage, gender and age were extracted. Subsequently, four variables including HPRS, stage, gender and age were submitted for recursive partitioning analysis to build a survival decision tree to optimize the risk stratification. As shown in the decision tree (Figure 6A), three different risk subgroups were defined based on two major components including HPRS as the most powerful parameter together with age (64 years old was identified as the cut-off point in the HPRS-high branch). Patients with low HPRS were defined as “low-risk” group, while “intermediate-risk” and “high-risk” groups were labelled with “High HPRS & Age < 64” and “High HPRS & Age ≥ 64”, respectively. Significant differences of overall survival were observed among the three risk subgroups (*P* = 3.95e-13; Figure 6B).

With a goal of quantifying the risk assessment for individual stage I patients, a nomogram was generated with HPRS together with other clinicopathological features, and the red arrow shows an example (Figure 6C). In the calibration analysis, the prediction lines of the nomogram for 3- and 5-year survival probability were extremely close to the ideal performance (45-degree line) (Figure 6D), indicating a high accuracy of the nomogram. When compared with other clinicopathological features, the nomogram exhibited the most powerful capacity for survival prediction (Figure 6E).

### Comprehensive analyses of enriched pathways and genomic alterations between different risk groups

**Figure 7:**
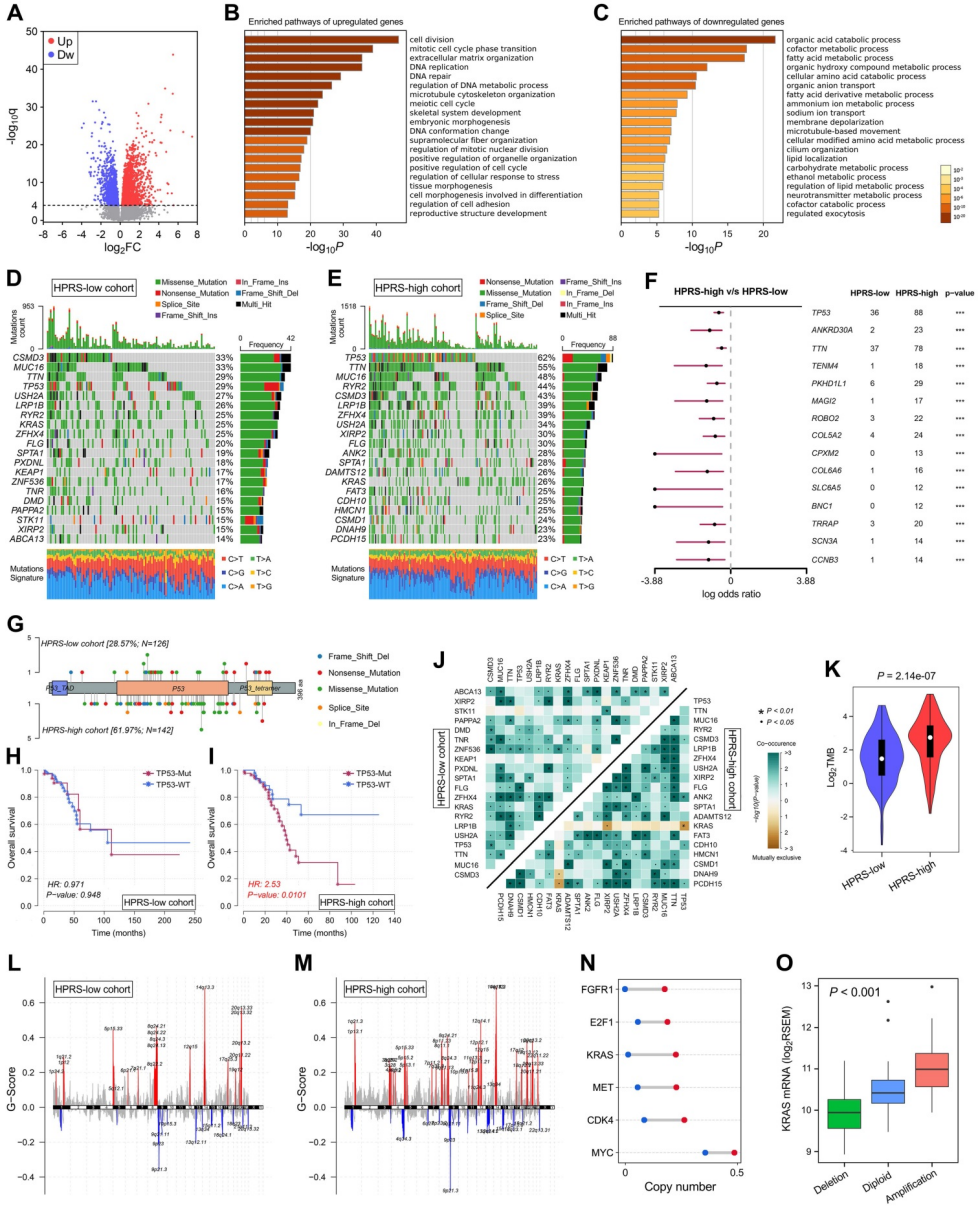
** Comprehensive analyses of enriched pathways and genomic alterations between different risk groups.** (A) Volcano plot shows DEGs between HPRS-low and -high groups in TCGA cohort. (B & C) Gene Ontology enrichment analysis was performed with significantly upregulated and downregulated genes, respectively. (D & E) Top 20 most frequently mutated genes were illustrated in each cohort. (F) TP53 occupies the top 1 position among differently mutated genes between HPRS-high and -low cohort. (G) A lollipop plot showed the different mutation spots of TP53 between two cohorts. (H & I) Kaplan-Meier analysis shows the independent relevance between overall survival and TP53 mutation in each cohort. (J) The heatmap illustrates the co-occurrence and mutually exclusive mutations of the top 25 frequently mutated genes in each cohort. (K) TMB was significantly elevated in the HPRS-high group. (L & M) Significant amplifications and deletions of copy number were detected and compared between the two cohorts. (N) Some representative oncogenes such as FGFR1, E2F1, KRAS, MET, CDK4 and MYC were widely amplified in the HPRS-high cohort compared to HPRS-low cohort. (O) KRAS is a typical example to demonstrate the positive correlation between copy number and mRNA expression in the HPRS-high cohort.

With a threshold of FDR q < 0.0001, 2,054 significantly upregulated genes and 1,579 significantly downregulated genes were identified in HPRS-high samples of TCGA cohort (Figure 7A). Subsequently, these DEGs were submitted to Metascape for Gene Ontology enrichment analysis. Upregulated genes were mainly enriched in pathways such as cell division, DNA repair and extracellular matrix organization (Figure 7B), while downregulated genes were mainly enriched in various metabolic processes (Figure 7C).

Top 20 most frequently mutated genes in each cohort were illustrated in Figure 7D & E. With a threshold of *P* value < 0.001 using Fisher's exact test, differently mutated genes were detected between the HPRS-high and -low cohort. Interestingly, TP53 occupies the top 1 position (Figure 7F), which suggests a high correlation with hypoxic condition in stage I patients. A lollipop plot revealed the different mutation spots of TP53 between two cohorts (Figure 7G), and the plausible chain reaction in survival difference was observed (Figure 7H & I). Furthermore, co-occurrence and mutually exclusive mutations were investigated, and a unique case of KRAS-TP53 mutually exclusive mutation was observed in HPRS-high cohort (Figure 7J), which indicates a probably common effect induced by their respective mutation and the selective advantages to keep more than one copy of the mutations. Moreover, the tumour mutational burden (TMB) was significantly elevated in the HPRS-high group (*P* = 2.14e-07; Figure 7K).

After removing germline CNV, significant amplifications and deletions were detected with a threshold of FDR < 0.05 in each cohort. By comparison, we observed more regions were altered in HPRS-high cohort (Figure 7L & M). In detail, some representative oncogenes such as FGFR1, E2F1, KRAS, MET, CDK4 and MYC were widely amplified in the HPRS-high cohort compared to HPRS-low cohort (Figure 7N). Furthermore, KRAS is a typical example to demonstrate the positive correlation between copy number and mRNA expression in the HPRS-high cohort (Figure 7O).

### Hypoxia-derived signatures predict therapeutic response in stage I patients

**Figure 8:**
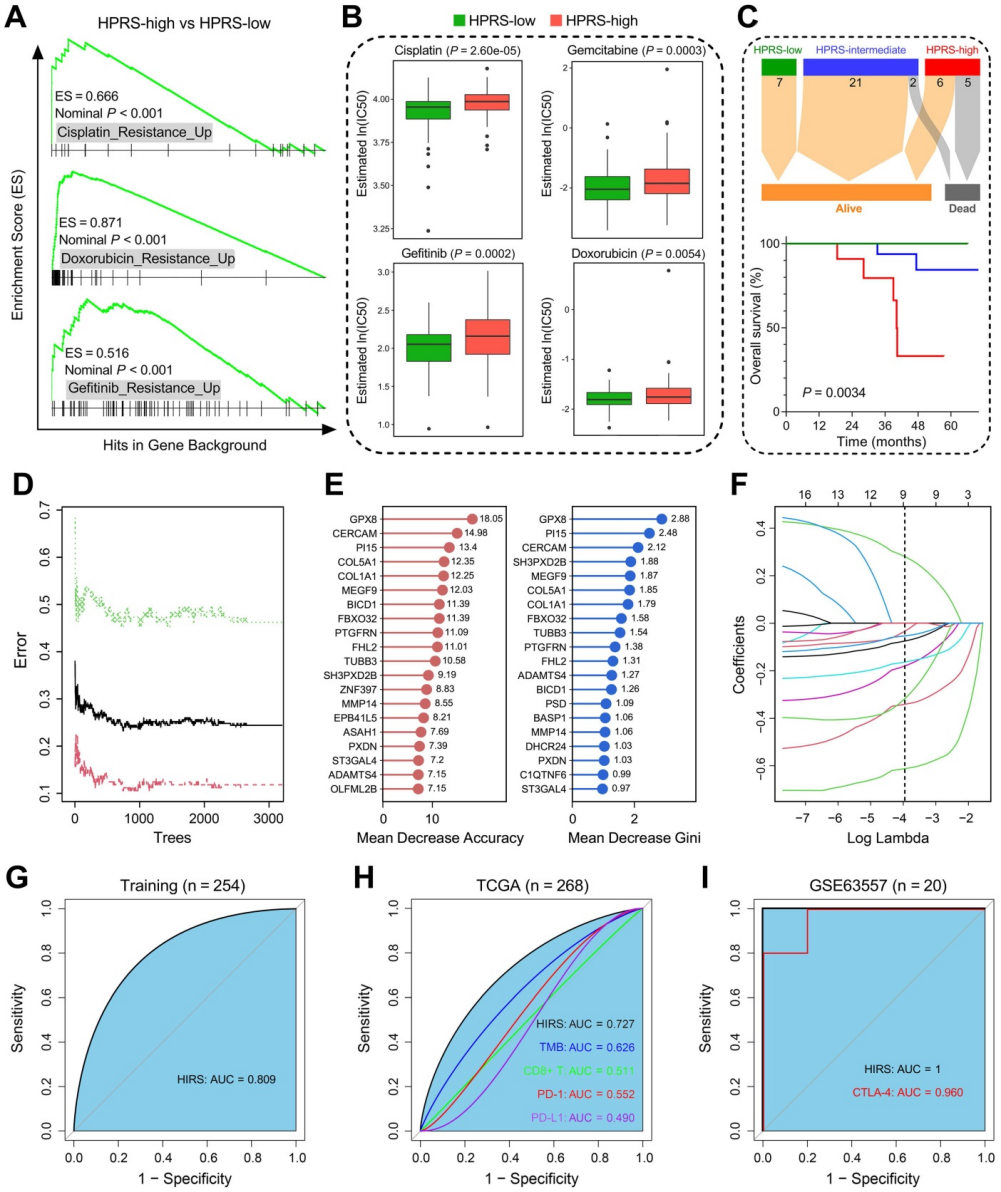
** Hypoxia-derived signatures predict therapeutic response in stage I patients.** (A) GSEA predicted that high HPRS is positively correlated with drug resistance in the training cohort. (B) Chemotherapeutic sensitivity of four common drugs (cisplatin, gemcitabine, gefitinib and doxorubicin) were estimated and compared in the training cohort. (C) Significant differences of overall survival were observed among different HPRS groups from 41 stage IB patients who received adjuvant chemotherapy. (D) The RF algorithm was used to screen for the most important genes associated with ICB therapy response. (E) 16 genes were overlapped in two ranking methods. (F) LASSO logistic regression analysis was further used to construct a robust signature to predict immunotherapeutic response. (G) HIRS exhibited the AUC of 0.809 to predict immunotherapeutic response in the training set. (H) In the testing TCGA cohort, HIRS showed the highest AUC of 0.727 compared to other common biomarkers for immunotherapy. (I) HIRS could completely discriminate responders and non-responders (AUC = 1) in 20 BALB/c mice subcutaneously inoculated with AB1-HA cells received anti-CTLA-4 treatment, even outperformed CTLA-4 expression (AUC = 0.960).

Based on the altered gene sets of different drug treatments retrieved from MSigDB, GSEA predicted that high HPRS is significantly correlated with drug resistance in the training cohort (Figure 8A). The R package 'pRRophetic' was used to estimate the chemotherapeutic sensitivity of four common drugs (cisplatin, gemcitabine, gefitinib and doxorubicin) used in LUAD treatment. The estimated IC50 values of these drugs were significantly elevated in HPRS-high samples of the training cohort (Figure 8B). Considering the controversial issue of the chemotherapeutic benefit in stage IB patients, we screened the six cohorts and extracted a total of 41 stage IB patients who received adjuvant chemotherapy and divided them into three groups according to their HPRS Z-score. Significant differences of overall survival were observed among different HPRS groups, indicating HPRS could serve as a promising biomarker to guide adjuvant chemotherapy in stage IB patients (Figure 8C).

Considering that hypoxia influences the efficacy of immunotherapy for cancer patients, we developed another hypoxia-derived gene signature to predict ICB therapy response using RF and LASSO logistic algorithms. With optimal values of two parameters (mtry = 133, ntree = 3,000; Figure 8D), the 199 stage I LUAD-specific hypoxia-related candidates were ranked according to their importance associated with immunotherapeutic response, and 16 genes were overlapped in two ranking methods (Figure 8E). Subsequently, in the LASSO logistic regression analysis, 10-fold cross-validation was applied to overcome over-fitting effect (Figure S4A), and an optimal λ value of 0.0188 was selected (Figure 8F). Nine genes (MEGF9, BICD1, TUBB3, ADAMTS4, PI15, FBXO32, ST3GAL4, GPX8, CERCAM) finally remained with individual coefficients (Figure S4B). In the training cohort, HIRS exhibited the AUC of 0.809 (Figure 8G) to predict immunotherapeutic response. In the testing TCGA cohort, HIRS showed the highest AUC of 0.727 compared to some common biomarkers for immunotherapy including TMB, CD8+ T cell infiltration, PD-1 and PD-L1 expression (Figure 8H). Surprisingly, HIRS could completely discriminate responders and non-responders (AUC = 1; Figure 8I) in 20 BALB/c mice subcutaneously inoculated with AB1-HA cells received anti-CTLA-4 treatment, even outperformed CTLA-4 expression (AUC = 0.960; Figure 8I).

## Discussion

Hypoxia is a common feature existing in most solid tumours 11. The hypoxic environment is a result of the imbalance between increased oxygen demand and insufficient oxygen supply, which is associated with a high proliferative rate in tumour 43. Hypoxia has a wide-ranging impact on various biological processes, such as metabolism, angiogenesis and metastasis 12, 44, 45. On the cellular level, hypoxia evokes a complex molecular response mainly dependent on the central role of transcription factor HIF1A 46. In solid tumours, crosstalk between hypoxia and other cancer-related hallmarks and pathways contributes to aggressive phenotypes and therapeutic resistance, which might lead to treatment failure and poor clinical outcome 11. These observations seem to explain why hypoxia is becoming an emerging biomarker and target in cancer therapy 47.

So far, some hypoxia gene signatures have been established for survival prediction in different cancer types including head and neck, prostate, bladder and breast cancer 48-51. Some studies established their gene signatures by testing candidate genes which were collected from previously published literatures, or used one single evaluative method such as Kaplan-Meier analysis to discriminate high-risk subset in the total patient cohort 48, 50. However, taking the fact into account that hypoxia is a complex process which involves regulation networks of many different genes, we established a hypoxia-related gene signature to predict survival in stage I-II LUAD patients in our previous study 14. Nevertheless, the relationships between hypoxia with clinical outcomes, oncogenic pathways, genomic alterations and therapeutic responses remain obscure in absolute early-stage (stage I) LUAD.

In this study, as many as possible (a total of 1,400) stage I LUAD patients were collected from 14 public datasets. Among various hallmarks of cancer, hypoxia was identified as a dominant risk factor in stage I LUAD. WGCNA was performed to identify a stage I LUAD-specific hypoxia-related co-expression network based on transcriptome profiling data. Considering HIF1A as a pivotal regulator in hypoxia, we intersected the “hypoxia-related module” with HIF1A targets in LUAD and a total of 199 stage I LUAD-specific hypoxia-related genes were identified. Next, we validated the 199 candidate genes in three aspects, including tumour tissues, single cells and cell lines. LASSO Cox regression model was used to screen for the most robust biomarkers to establish a prognostic signature, and a formula for calculation of hypoxia-related prognostic risk score (HPRS) was established. Subsequently, the prognostic capacity was validated in five independent cohorts across different platforms. Notably, in the pooled cohort of stage I patients, we observed that HPRS exerted a more significant risk on overall survival than pathological stage (IB vs IA), indicating there is an urgent need to introduce molecular classification into tumour staging. Even in the stage IA patients with tumour size smaller than 2 cm, HPRS retained its prognostic capacity to discriminate the high-risk subset, which might benefit from adjuvant chemotherapy. Enriched pathways, genomic alterations and CNVs were also analysed and compared in different HPRS groups, and we observed that high HPRS was significantly correlated with more aggressive molecular changes such as TP53 mutation and amplifications of driver oncogenes. These genomic alterations drive rapid proliferative rate by consuming oxygen as well as generating aberrant vasculature at the early stage of tumor progression. Especially, mutant p53 cooperates with HIF-1 in transcriptional regulation of a specific subset of pro-tumorigenic genes to induce hypoxic condition and thus to promote NSCLC progression 52. On the basis of aforementioned findings, intratumoral hypoxia seemed to be a major cancer hallmark, associated with the worse survival in stage I LUAD patients.

A survival decision tree was built to improve risk stratification based on HPRS, age, gender and pathological stage for stage I patients. Only two components remained in the decision tree: HPRS acted as the major determinant, and older age (64 years as cut-off point) the secondary. Furthermore, a nomogram was generated to quantify risk assessment and survival probability. Compared to other traditional features, the nomogram exhibited the highest accuracy and discrimination in survival prediction.

Reliable biomarkers to predict immunotherapeutic responses in stage I LUAD remain unmet in clinical practice. Accumulating evidence have demonstrated that hypoxia influences the efficacy of immunotherapy 53. Thus, we developed a hypoxia-related immunotherapeutic response score (HIRS) to predict ICB therapy response for stage I patients using RF and LASSO logistic algorithms. The utility of HIRS was further validated in different cohorts, even outperformed conventional immunotherapy biomarkers.

The retrospective nature of our study is an inevitable limitation. Although we included as many datasets as possible for rigorous validation, and different approaches such as RMA and Combat were combined to reduce batch effects, we have to acknowledge the fact that sampling bias caused by tumour genetic heterogeneity and cross-platform integration could only be reduced, but not completely eliminated. Meanwhile, further experimental studies are expected to elucidate tumour hypoxia-related biological functions underlying the gene signature in LUAD.

In this study, we identified hypoxia as a primary prognostic risk factor for stage I LUAD among all the cancer-related hallmarks. Two hypoxia-related signatures were established to predict overall survival and immunotherapeutic response for stage I LUAD patients, respectively. A survival decision tree was built to optimize risk stratification, and a nomogram was generated to quantify risk assessment. The enriched pathways, genomic alterations and CNVs were analysed and compared between different risk groups. In summary, our study might provide some useful clues for introducing molecular classification into tumour staging and guiding treatment decision-making, finally promoting personalized management of stage I LUAD.

## Supplementary Material

Supplementary figures and table.

## Abbreviations

TNM: tumour-node-metastasis
NSCLC: non-small cell lung cancer
LUAD: lung adenocarcinoma
GEO: Gene Expression Omnibus
TCGA: The Cancer Genome Atlas
CCLE: Cancer Cell Line Encyclopedia
RMA: robust multi-array average
ssGSEA: single-sample gene set enrichment analysis
WGCNA: weighted gene co-expression network analysis
LASSO: least absolute shrinkage and selection operator
RF: random forest
HPRS: hypoxia-related prognostic risk score
HIRS: hypoxia-related immunotherapeutic response score
NMF: non-negative matrix factorization
PCA: principal component analysis
HR: hazard ratio
C-index: concordance index
DCA: decision curve analysis
TMB: tumour mutational burden
CNV: copy number variation
ICB: immune checkpoint blockade
TIDE: tumour immune dysfunction and exclusion
