# Supplementary material for: Identification and validation of hypoxia-derived gene signatures to predict clinical outcomes and therapeutic responses in stage I lung adenocarcinoma patients
Source: Theranostics. 2021 Mar 5;11(10):5061–76. doi: 10.7150/thno.56202 (PMC7978303; doi:10.7150/thno.56202)
Supplement: Supplementary file 1 — Supplementary figures and table. [file thnov11p5061s1.pdf]

Supplementary files

Identification and validation of hypoxia-derived gene signatures to predict clinical outcomes and therapeutic responses in stage I lung adenocarcinoma patients

Run Shi[1,†], Xuanwen Bao[2,†], Kristian Unger[1,3], Jing Sun[1], Shun Lu[4], Farkhad Manapov[1], Xuanbin Wang[5], Claus Belka[1], Minglun Li[1,#]

1. Department of Radiation Oncology, University Hospital, LMU Munich, Munich D-81377, Germany.
2. Department of Medical Oncology, The First Affiliated Hospital, College of Medicine, Zhejiang University, Hangzhou 310003, China.
3. Research Unit Radiation Cytogenetics, Helmholtz Center Munich, German Research Center for Environmental Health GmbH, Neuherberg D-85764, Germany.
4. Department of Radiotherapy, Sichuan Cancer Hospital, School of Medicine, University of Electronic Science and Technology of China, Chengdu 610041, China.
5. Laboratory of Chinese Herbal Pharmacology, Oncology Center, Renmin Hospital, Hubei Key Laboratory of Wudang Local Chinese Medicine Research, Hubei University of Medicine, Shiyan 442000, China.

† These authors contributed equally to this work.

# Corresponding author.

Dr. med. Minglun Li

Department of Radiation Oncology, University Hospital, LMU Munich, Marchioninistr. 15, D-81377 Munich, Germany.

Tel: (089) 4400-73770

Fax: (089) 4400-76770

E-mail: minglun.li@med.uni-muenchen.de

Clinicopathological Features of Patients in Each Cohort

	Training set	Validation I	Validation II	Validation III	Validation IV	Validation V
Platform	Rosetta/Merck Human RSTA Custom Affymetrix 2.0 microarray	Affymetrix Human Genome U133A Array	Affymetrix Human Genome U133 Plus 2.0 Array	Illumina HiSeq 2000 RNA Sequencing	Illumina HumanWG-6 v3.0 expression beadchip	Agilent-014850 Whole Human Genome Microarray 4x44K G4112F
Component	GSE72094	GSE68465<br>GSE14814<br>GSE31547<br>Chitale's cohort	GSE30219<br>GSE31210<br>GSE50081<br>GSE37745<br>GSE29013<br>E-MTAB-923	TCGA	GSE41271	GSE13213
No. of samples	254	226	471	269	101	79
Median age (range)	70 (38-89)	63 (35-87)**	63 (34-86)	67 (38-88)	64 (42-83)	61 (35-84)
Female (%)	139 (54.7)	133 (58.8)	250 (53.1)	158 (58.7)	56 (55.4)	38 (48.1)
Male (%)	115 (45.3)	93 (41.2)	221 (46.9)	111 (41.3)	45 (44.6)	41 (51.9)
Stage I (%)*	5 (2.0)	14 (6.2)	0 (0)	5 (1.9)	0 (0)	0 (0)
Stage IA (%)	150 (59.0)	143 (63.3)	280 (59.4)	130 (48.3)	37 (36.6)	42 (53.2)
Stage IB (%)	99 (39.0)	69 (30.5)	191 (40.6)	134 (49.8)	64 (63.4)	37 (46.8)
Median follow-up (months)	28.45	40	63.41	23.37	45.93	68.9
No. of death (%)	52 (20.5)	63 (27.9)	146 (31.0)	65 (24.2)	27 (26.7)	25 (31.6)

* annotated as stage I patients only

** age is not available in Chitale's cohort (n=53)

Figure S1: Summarization of clinicopathological features in each cohort.

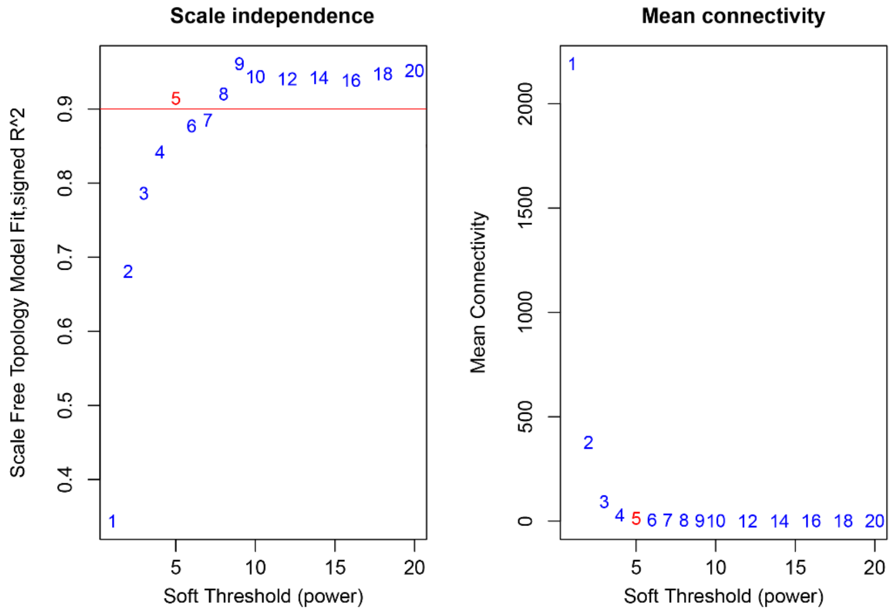

Figure S2: A power of β=5 was chosen as the optimal soft threshold to ensure a scale-free co-expression network.

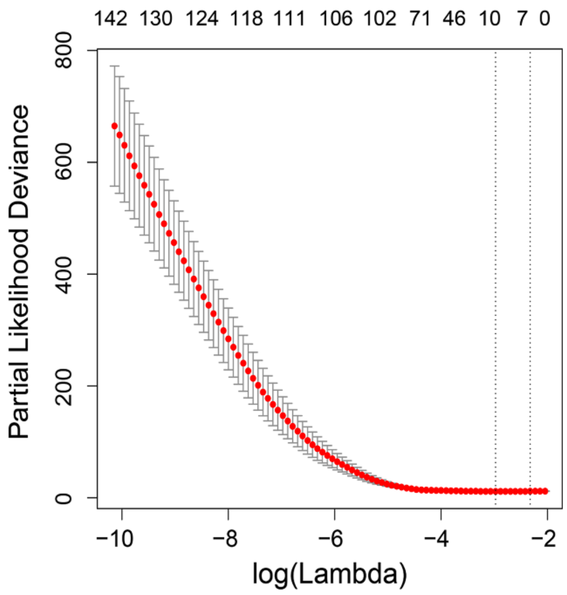

Figure S3: In the LASSO Cox regression model, 10-fold cross-validation was applied to overcome over-fitting effect, and an optimal λ value of 0.051 was selected.

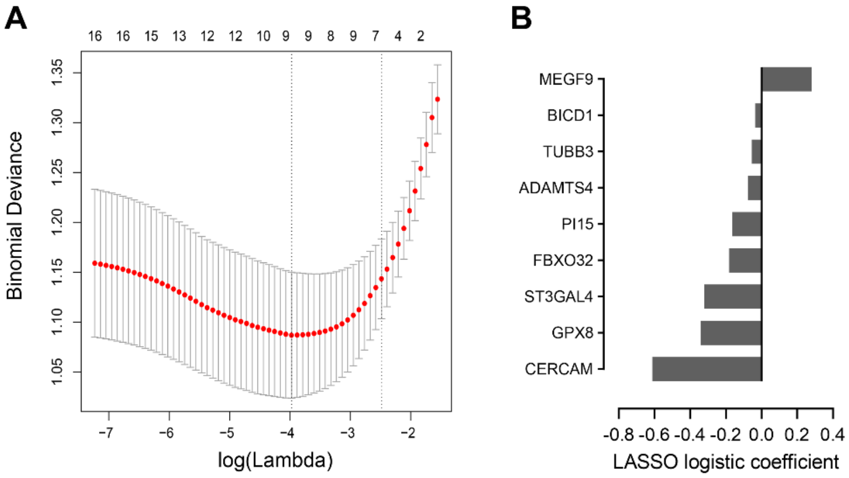

Figure S4: LASSO logistic regression analysis. (A) 10-fold cross-validation was applied to overcome over-fitting effect, and an optimal λ value of 0.0188 was selected. (B) Distribution of individual coefficients of the HIRS signature.

Table S1: Details of 199 stage I LUAD-specific hypoxia-related candidate genes.

Official gene symbol	EntrezGene ID	Name
ABCD3	5825	ATP binding cassette subfamily D member 3(ABCD3)
ABL2	27	ABL proto-oncogene 2, non-receptor tyrosine kinase(ABL2)
ACSS2	55902	acyl-CoA synthetase short-chain family member 2(ACSS2)
ADAMTS4	9507	ADAM metallopeptidase with thrombospondin type 1 motif 4(ADAMTS4)
AFAP1	60312	actin filament associated protein 1(AFAP1)
AHCYL2	23382	adenosylhomocysteinase like 2(AHCYL2)
APOM	55937	apolipoprotein M(APOM)
AQP7	364	aquaporin 7(AQP7)
ARL10	285598	ADP ribosylation factor like GTPase 10(ARL10)
ASAH1	427	N-acylsphingosine amidohydrolase 1(ASAH1)
ASAP1	50807	ArfGAP with SH3 domain, ankyrin repeat and PH domain 1(ASAP1)
ASB3	51130	ankyrin repeat and SOCS box containing 3(ASB3)
ATP11A	23250	ATPase phospholipid transporting 11A(ATP11A)
ATP1A1	476	ATPase Na+/K+ transporting subunit alpha 1(ATP1A1)
BASP1	10409	brain abundant membrane attached signal protein 1(BASP1)
BCAM	4059	basal cell adhesion molecule (Lutheran blood group)(BCAM)
BCKDHA	593	branched chain keto acid dehydrogenase E1, alpha polypeptide(BCKDHA)
BCKDHB	594	branched chain keto acid dehydrogenase E1 subunit beta(BCKDHB)
BCL2L11	10018	BCL2 like 11(BCL2L11)
BICD1	636	BICD cargo adaptor 1(BICD1)
C10orf55	414236	chromosome 10 open reading frame 55(C10orf55)
C11orf1	64776	chromosome 11 open reading frame 1(C11orf1)
C11orf49	79096	chromosome 11 open reading frame 49(C11orf49)
C11orf54	28970	chromosome 11 open reading frame 54(C11orf54)
C16orf72	29035	chromosome 16 open reading frame 72(C16orf72)
C1orf210	149466	chromosome 1 open reading frame 210(C1orf210)
C1QTNF6	114904	C1q and tumor necrosis factor related protein 6(C1QTNF6)
CALU	813	calumenin(CALU)
CASD1	64921	CAS1 domain containing 1(CASD1)
CD109	135228	CD109 molecule(CD109)
CERCAM	51148	cerebral endothelial cell adhesion molecule(CERCAM)
CHADL	150356	chondroadherin like(CHADL)
CHST15	51363	carbohydrate sulfotransferase 15(CHST15)
CLK3	1198	CDC like kinase 3(CLK3)
COL12A1	1303	collagen type XII alpha 1 chain(COL12A1)
COL1A1	1277	collagen type I alpha 1 chain(COL1A1)
COL5A1	1289	collagen type V alpha 1 chain(COL5A1)

COL6A3	1293	collagen type VI alpha 3 chain(COL6A3)
CPD	1362	carboxypeptidase D(CPD)
CRYM	1428	crystallin mu(CRYM)
CTHRC1	115908	collagen triple helix repeat containing 1(CTHRC1)
CUL4B	8450	cullin 4B(CUL4B)
CXorf23	256643	chromosome X open reading frame 23(CXorf23)
DCXR	51181	dicarbonyl and L-xylulose reductase(DCXR)
DHCR24	1718	24-dehydrocholesterol reductase(DHCR24)
DSC2	1824	desmocollin 2(DSC2)
DYRK2	8445	dual specificity tyrosine phosphorylation regulated kinase 2(DYRK2)
ENTPD7	57089	ectonucleoside triphosphate diphosphohydrolase 7(ENTPD7)
EPB41L5	57669	erythrocyte membrane protein band 4.1 like 5(EPB41L5)
EVPLL	645027	envoplakin like(EVPLL)
EXOC8	149371	exocyst complex component 8(EXOC8)
FAAH	2166	fatty acid amide hydrolase(FAAH)
FAM171B	165215	family with sequence similarity 171 member B(FAM171B)
FAM3C	10447	family with sequence similarity 3 member C(FAM3C)
FAP	2191	fibroblast activation protein alpha(FAP)
FBXO32	114907	F-box protein 32(FBXO32)
FCHO1	23149	FCH domain only 1(FCHO1)
FHL2	2274	four and a half LIM domains 2(FHL2)
FKBP14	55033	FK506 binding protein 14(FKBP14)
FN1	2335	fibronectin 1(FN1)
FOSL1	8061	FOS like 1, AP-1 transcription factor subunit(FOSL1)
FRAT1	10023	frequently rearranged in advanced T-cell lymphomas 1(FRAT1)
FRMD6	122786	FERM domain containing 6(FRMD6)
FRS3	10817	fibroblast growth factor receptor substrate 3(FRS3)
FZD5	7855	frizzled class receptor 5(FZD5)
GJB2	2706	gap junction protein beta 2(GJB2)
GLYR1	84656	glyoxylate reductase 1 homolog(GLYR1)
GNMT	27232	glycine N-methyltransferase(GNMT)
GPC6	10082	glypican 6(GPC6)
GPR160	26996	G protein-coupled receptor 160(GPR160)
GPRC5C	55890	G protein-coupled receptor class C group 5 member C(GPRC5C)
GPX8	493869	glutathione peroxidase 8 (putative)(GPX8)
GRAMD2	196996	GRAM domain containing 2(GRAMD2)
HAUS7	55559	HAUS augmin like complex subunit 7(HAUS7)
HEMK1	51409	HemK methyltransferase family member 1(HEMK1)
HPN	3249	hepsin(HPN)
HS2ST1	9653	heparan sulfate 2-O-sulfotransferase 1(HS2ST1)
IKBIP	121457	IKBKB interacting protein(IKBIP)
ITGB3	3690	integrin subunit beta 3(ITGB3)
ITPR3	3710	inositol 1,4,5-trisphosphate receptor type 3(ITPR3)

KDELC1	79070	KDEL motif containing 1(KDELC1)
KDM6A	7403	lysine demethylase 6A(KDM6A)
KIAA0232	9778	KIAA0232(KIAA0232)
KIF3C	3797	kinesin family member 3C(KIF3C)
KLF7	8609	Kruppel like factor 7(KLF7)
KLHDC9	126823	kelch domain containing 9(KLHDC9)
KLK6	5653	kallikrein related peptidase 6(KLK6)
KPNA4	3840	karyopherin subunit alpha 4(KPNA4)
LCN12	286256	lipocalin 12(LCN12)
LIPH	200879	lipase H(LIPH)
LMO7	4008	LIM domain 7(LMO7)
LOX	4015	lysyl oxidase(LOX)
LOXL2	4017	lysyl oxidase like 2(LOXL2)
MAPK6	5597	mitogen-activated protein kinase 6(MAPK6)
MEGF9	1955	multiple EGF like domains 9(MEGF9)
MFI2	4241	melanotransferrin(MFI2)
MMAB	326625	methylmalonic aciduria (cobalamin deficiency) cblB type(MMAB)
MMD	23531	monocyte to macrophage differentiation associated(MMD)
MMP14	4323	matrix metallopeptidase 14(MMP14)
MTMR11	10903	myotubularin related protein 11(MTMR11)
MVK	4598	mevalonate kinase(MVK)
MYO1E	4643	myosin IE(MYO1E)
MYO6	4646	myosin VI(MYO6)
NAMPT	10135	nicotinamide phosphoribosyltransferase(NAMPT)
NAV1	89796	neuron navigator 1(NAV1)
NBEAL1	65065	neurobeachin like 1(NBEAL1)
NEK8	284086	NIMA related kinase 8(NEK8)
NRIP1	8204	nuclear receptor interacting protein 1(NRIP1)
NUBP1	4682	nucleotide binding protein 1(NUBP1)
OLFML2B	25903	olfactomedin like 2B(OLFML2B)
OSBPL9	114883	oxysterol binding protein like 9(OSBPL9)
PCP2	126006	Purkinje cell protein 2(PCP2)
PGAP2	27315	post-GPI attachment to proteins 2(PGAP2)
PGM2L1	283209	phosphoglucomutase 2 like 1(PGM2L1)
PI15	51050	peptidase inhibitor 15(PI15)
PIGA	5277	phosphatidylinositol glycan anchor biosynthesis class A(PIGA)
PLA2G6	8398	phospholipase A2 group VI(PLA2G6)
PLAU	5328	plasminogen activator, urokinase(PLAU)
PLAUR	5329	plasminogen activator, urokinase receptor(PLAUR)
PLEKHB1	58473	pleckstrin homology domain containing B1(PLEKHB1)
PLIN3	10226	perilipin 3(PLIN3)
PLOD2	5352	procollagen-lysine,2-oxoglutarate 5-dioxygenase 2(PLOD2)
PMM1	5372	phosphomannomutase 1(PMM1)

PNKD	25953	paroxysmal nonkinesigenic dyskinesia(PNKD)
POLR3H	171568	RNA polymerase III subunit H(POLR3H)
PPL	5493	periplakin(PPL)
PRR15L	79170	proline rich 15 like(PRR15L)
PSD	5662	pleckstrin and Sec7 domain containing(PSD)
PTGFRN	5738	prostaglandin F2 receptor inhibitor(PTGFRN)
PTS	5805	6-pyruvoyltetrahydropterin synthase(PTS)
PXDN	7837	peroxidasin(PXDN)
PXMP4	11264	peroxisomal membrane protein 4(PXMP4)
RAB17	64284	RAB17, member RAS oncogene family(RAB17)
RAB40B	10966	RAB40B, member RAS oncogene family(RAB40B)
RASAL2	9462	RAS protein activator like 2(RASAL2)
RASSF7	8045	Ras association domain family member 7(RASSF7)
RBBP9	10741	RB binding protein 9, serine hydrolase(RBBP9)
REV1	51455	REV1, DNA directed polymerase(REV1)
RPL37	6167	ribosomal protein L37(RPL37)
RPRD2	23248	regulation of nuclear pre-mRNA domain containing 2(RPRD2)
SCAI	286205	suppressor of cancer cell invasion(SCAI)
SCARB2	950	scavenger receptor class B member 2(SCARB2)
SEC23A	10484	Sec23 homolog A, coat complex II component(SEC23A)
SEPW1	6415	selenoprotein W(SEPW1)
SERPINE1	5054	serpin family E member 1(SERPINE1)
SFT2D3	84826	SFT2 domain containing 3(SFT2D3)
SGSM1	129049	small G protein signaling modulator 1(SGSM1)
SH3PXD2B	285590	SH3 and PX domains 2B(SH3PXD2B)
SH3RF1	57630	SH3 domain containing ring finger 1(SH3RF1)
SIRT3	23410	sirtuin 3(SIRT3)
SLC16A1	6566	solute carrier family 16 member 1(SLC16A1)
SLC25A38	54977	solute carrier family 25 member 38(SLC25A38)
SLC39A14	23516	solute carrier family 39 member 14(SLC39A14)
SNRNP200	23020	small nuclear ribonucleoprotein U5 subunit 200(SNRNP200)
SOX6	55553	SRY-box 6(SOX6)
SPHK1	8877	sphingosine kinase 1(SPHK1)
ST3GAL4	6484	ST3 beta-galactoside alpha-2,3-sialyltransferase 4(ST3GAL4)
ST7	7982	suppression of tumorigenicity 7(ST7)
STC1	6781	stanniocalcin 1(STC1)
STK38L	23012	serine/threonine kinase 38 like(STK38L)
STOX2	56977	storkhead box 2(STOX2)
STX19	415117	syntaxin 19(STX19)
STX6	10228	syntaxin 6(STX6)
STXBP5	134957	syntaxin binding protein 5(STXBP5)
SUCLG2	8801	succinate-CoA ligase GDP-forming beta subunit(SUCLG2)
SULF2	55959	sulfatase 2(SULF2)

SUOX	6821	sulfite oxidase(SUOX)
SYNJ2	8871	synaptojanin 2(SYNJ2)
TAB3	257397	TGF-beta activated kinase 1/MAP3K7 binding protein 3(TAB3)
TCEA3	6920	transcription elongation factor A3(TCEA3)
TJP2	9414	tight junction protein 2(TJP2)
TMCC1	23023	transmembrane and coiled-coil domain family 1(TMCC1)
TMEM185B	79134	transmembrane protein 185B(TMEM185B)
TMEM205	374882	transmembrane protein 205(TMEM205)
TMEM63B	55362	transmembrane protein 63B(TMEM63B)
TNFRSF21	27242	TNF receptor superfamily member 21(TNFRSF21)
TNFSF15	9966	tumor necrosis factor superfamily member 15(TNFSF15)
TNFSF4	7292	tumor necrosis factor superfamily member 4(TNFSF4)
TRDMT1	1787	tRNA aspartic acid methyltransferase 1(TRDMT1)
TRIP12	9320	thyroid hormone receptor interactor 12(TRIP12)
TRNAU1AP	54952	tRNA selenocysteine 1 associated protein 1(TRNAU1AP)
TRPV3	162514	transient receptor potential cation channel subfamily V member 3(TRPV3)
TSTD1	100131187	thiosulfate sulfurtransferase like domain containing 1(TSTD1)
TTYH3	80727	tweety family member 3(TTYH3)
TUBB3	10381	tubulin beta 3 class III(TUBB3)
TXLNG	55787	taxilin gamma(TXLNG)
TXNRD2	10587	thioredoxin reductase 2(TXNRD2)
UNC13B	10497	unc-13 homolog B(UNC13B)
UQCR10	29796	ubiquinol-cytochrome c reductase, complex III subunit X(UQCR10)
VAMP8	8673	vesicle associated membrane protein 8(VAMP8)
VANGL1	81839	VANGL planar cell polarity protein 1(VANGL1)
VCAN	1462	versican(VCAN)
WISP1	8840	WNT1 inducible signaling pathway protein 1(WISP1)
XIRP1	165904	xin actin binding repeat containing 1(XIRP1)
ZNF33B	7582	zinc finger protein 33B(ZNF33B)
ZNF397	84307	zinc finger protein 397(ZNF397)
ZNF76	7629	zinc finger protein 76(ZNF76)
ZSWIM5	57643	zinc finger SWIM-type containing 5(ZSWIM5)
ZSWIM7	125150	zinc finger SWIM-type containing 7(ZSWIM7)
